# Characteristic analysis of the chloroplast genome of *Cyathula capitata* and comparative analysis with *Cyathula officinalis* and their hybrid *Cyathula officinalis × Cyathula capitata*

**DOI:** 10.3389/fpls.2026.1772997

**Published:** 2026-03-04

**Authors:** Bixing Gao, Yisha Zeng, Min Tang, Maohua Yuan, Ke Deng, Yan Gou, Guihua Jiang

**Affiliations:** 1 Industrial Technology Basic Public Service Platform, Ministry of Industry and Information Technology, Sichuan Institute for Drug Control (Sichuan Medical Device Testing Center, Sichuan Musk Deer Research Institute), Chengdu, China; 2 College of Pharmacy, Chengdu University of Traditional Chinese Medicine, Chengdu, China; 3 Department of New Energy Materials and Chemistry, Leshan Normal University, Leshan, China

**Keywords:** chloroplast genome, *Cyathula capitata*, *Cyathula officinalis*, phylogenetic analysis, sequence characteristic

## Abstract

**Introduction:**

*Cyathula capitata* (Wall.) Moq., a widely used medicinal herb in Yi medicine, is often combined with other herbs to treat traumatic injuries, rheumatism, and rheumatoid arthritis. This study aims to characterize the chloroplast genomes, assess variation levels, and elucidate the phylogenetic relationships among *Cyathula capitata*, *Cyathula officinalis*, and their hybrid (*Cyathula officinalis* × *Cyathula capitata*). The findings provide valuable references for species identification, genetic background analysis, and quality control of Cyathula medicinal materials.

**Methods:**

We employed second-generation high-throughput sequencing technology to sequence the chloroplast genomes of *Cyathula capitata*, *Cyathula officinalis*, and their hybrid (*Cyathula officinalis* × *Cyathula capitata*). Comparative genomic analyses were conducted to examine their genomic structures, simple sequence repeats (SSRs), codon usage preferences, and inverted repeat (IR) regions. Additionally, a phylogenetic tree based on chloroplast genome sequences was constructed to clarify their evolutionary relationships.

**Results:**

The chloroplast genomes of all three taxa displayed the typical quadripartite structure, comprising a large single-copy region, a small single-copy region, and two IR regions (IRa and IRb). The *Cyathula capitata* genomes ranged from 151,428 to 151,436 bp, showing notable intraspecific diversity likely influenced by geographic factors. However, two *C. capitata* samples shared an identical genome length of 151,518 bp and exhibited consistent genotypes across locations, indicating relative genomic conservation. Repeat sequence analysis identified hexanucleotide SSRs, a unique 16-bp single nucleotide insertion in the rpl22 gene, and a 30–40 bp forward repeat within the rps16_1-trnQ-UUG intergenic region as distinguishing markers for *Cyathula officinalis* and its hybrids. Codon usage analysis revealed no strong bias at the third codon position among the three species, although codons ending with thymine (T) were used more frequently. IR boundary analysis showed variation only among isolated *C. capitata* samples. Comparative genomics highlighted psbI-trnS-GCU and rps16_1-trnQ-UUG as highly variable regions. Phylogenetic analysis indicated that the hybrid (Z4) grouped within *C. capitata*, consistent with the maternal inheritance pattern of chloroplast genomes. Given the potential for variations in maternal parents among different hybrid batches, the positions of these elements in the phylogenetic tree may accordingly vary.

**Conclusion:**

This study elucidated the chloroplast genome features and phylogenetic relationships of *C. capitata, C. officinalis*, and their hybrid. The findings offer significant molecular insights that facilitate species identification, genetic analysis, and quality assessment of Cyathula medicinal resources. These insights support the sustainable utilization and conservation of these resources.

## 1 Introduction

Naisibuni (ꇌꌦꁬꅪ), derived from the dried root of *Cyathula capitata* (Wall.) Moq., a species within the genus *Cyathula* of the Amaranthaceae family (Azi, 1994), is widely used in Yi medicine. Locally, it is known as Lebu or Naibu in the Liangshan Yi Autonomous Prefecture and is also referred to as Ma Niuxi or Ku Niuxi. The herb is characterized by a bitter, astringent, and slightly numbing taste, and is classified as neutral in nature (Li and He, 1990). Its primary functions include dispelling wind and dampness, removing blood stasis, and unblocking meridians (Rao, 2006). Commonly used in combination with other herbs, Naisibuni is primarily employed to treat “Sise”, a condition described as obstruction of qi and blood caused by the interaction of internal and external factors, and traumatic injuries. Clinical research has demonstrated that the Yi medicine formula “Wosi,” which features Naisibuni as its principal component, achieves high cure rates, low recurrence rates, and minimal adverse reactions, effectively addressing both symptoms and root causes in the treatment of gouty arthritis (GA) (Zhang et al., 2020; Zhou, 2020; Gu et al., 2024).

The medicinal plants “Naisibuni” (*Cyathula capitata*) and *C. officinalis* from the genus *Cyathula* (*Amaranthaceae*) have historically been well-documented in medicinal applications. However, due to the morphological similarities between the two source plants, they have long been prone to misidentification and confused use in the market. Conventional identification methods principally depend on morphological and histological characteristics, which are inherently subjective. Therefore, this study conducts a comparative analysis at the chloroplast genome level to provide molecular evidence for accurate identification.As stated in the “Niuxi Jie” section of Zhang Xichun’s Medical Records Combining Chinese and Western Medicine from the Republican Period, the following is documented: “Sichuan-produced ones exhibit two distinct colors, purple and white, with the purple ones being considered superior” (Zhang, 2016). When considered in conjunction with the description of plant morphology in the Flora of China (Flora of China Editorial Committee, 1979), it can be inferred that the *C. officinalis* utilized historically encompassed both *C. capitata* and *C. officinalis*, with Naisibuni being regarded as of superior quality. However, due to factors such as introduction and cultivation, resource changes, and market circulation, the primary botanical origin of modern commercial *C. officinalis* has gradually shifted to *C. officinalis* as recorded in the Chinese Pharmacopoeia, while Naisibuni (often referred to as “Maniuxi” in the market) is commonly circulated as a regional customary substitute or an adulterant. Additionally, the natural hybrid between these two species—hybrid *Cyathula* (*C. officinalis × C. capitata*)—is also present in the market, further complicating accurate identification (Shi et al., 2017). A thorough investigation has revealed that “Niuxi” medicinal materials exhibit a variety of nomenclature and complex origins (Gao et al., 2024). This investigation has identified issues such as “different materials under the same name” and “same material under different names.” It is worth noting that a common misrepresentation of Naisibuni is to refer to him as *C. officinalis*. Consequently, Naisibuni is frequently designated as a significant adulterant of *C. officinalis* in identification studies. However, Naisibuni has been shown to possess distinctive therapeutic efficacy in the treatment of conditions such as rheumatism and arthralgia. The utilization of morphological and histological methods for identification is subject to limitations inherent in the methods themselves. Therefore, this study investigates the chloroplast genomes of Naisibuni and *C. officinalis* to provide supplementary molecular evidence for their differentiation.

Chloroplasts are semiautonomous organelles unique to green plants and certain protists, capable of photosynthesis and containing their own independent genomes (Margulis and Goode, 1981). The chloroplast genome of higher plants typically exhibits a highly conserved quadripartite structure, with sizes ranging from 115 to 165 kilobases (Jansen et al., 2005). This circular genome is divided by a pair of IR sequences (IRs) into a large single-copy (LSC) region and a small single-copy (SSC) region (Qian et al., 2021). Most angiosperm chloroplast genomes contain approximately 120 to 160 distinct genes (Jianguo et al., 2018), encoding proteins involved primarily in photosynthesis and gene expression, alongside transfer RNA (tRNA) and ribosomal RNA (rRNA) genes. Due to its high degree of conservation, abundant genetic information, and predominantly maternal inheritance, the chloroplast genome serves as an ideal resource for studying plant genetic diversity and conducting phylogenetic analyses (McFadden, 2001; Olmstead and Palmer, 1994). Furthermore, with the rapid advancement of high-throughput sequencing technologies and the moderate nucleotide substitution rate of chloroplast DNA (cpDNA), which is significantly lower than that of nuclear DNA, the sequencing and assembly of chloroplast genomes have become increasingly efficient and accessible (Carbonell-Caballero et al., 2015). Consequently, chloroplast genome sequencing has emerged as a vital molecular marker approach for investigating plant phylogenetics and genetic differentiation.

Currently, *Cyathula officinalis* Kuan is frequently used as a substitute for *Cyathula capitata* in research. Adding to the complexity, hybrids between these two species, referred to as hybrid *Cyathula*, are commonly found in the market, further complicating the understanding of the genetic background and parental origins of *Cyathula* medicinal materials. Previous studies have compared the chloroplast genomes of *Cyathula officinalis* Kuan with those of Naisibuni and three other *Cyathula* species (Guo et al., 2022). However, molecular approaches specifically designed to identify the parental origins of hybrid *Cyathula* remain undeveloped. Moreover, significant intraspecific variation may occur within the same Naisibuni cultivar due to geographical differences (Fu et al., 2013). This study systematically compares the chloroplast genomes of *Cyathula capitata*, *Cyathula officinalis*, and their hybrid (*Cyathula officinalis* × *Cyathula capitata*) from diverse origins. It aims to elucidate the structural features and patterns of inter- and intraspecific variation within the chloroplast genomes, identify the maternal lineage of the hybrid, and infer parental preferences.

## 2 Materials and methods

### 2.1 Plant materials

Based on a comprehensive literature review and field survey, fresh plant samples were collected in July 2024, including three batches of *Cyathula capitata*, two batches of *Cyathula officinalis*, and six batches of their hybrid (*Cyathula officinalis* × *Cyathula capitata*). Young leaves were carefully selected, rinsed with clean water, and immediately dried using absorbent paper to remove surface moisture. The samples were then placed into cryogenic tubes and stored on dry ice for subsequent DNA extraction. Detailed records of sampling time, location, latitude, longitude, and altitude were maintained. Species identification of the original plants was confirmed by Professor Jiang Guihua from Chengdu University of Traditional Chinese Medicine and Senior Pharmacist Gao Bixing of the Sichuan Provincial Institute for Drug Control (**Supplementary Table 1**). Based on preliminary surveys, samples of *C. capitata* (M2, M3) were collected from Liangshan, Sichuan, where *C. officinalis* is absent, and samples of *C. officinalis* (C1) were obtained from Hubei, where *C. capitata* does not occur—representing allopatric populations without coexistence of the other parental species. Conversely, hybrid samples (Z1–Z6) and potential parental samples were collected from regions such as Ya’an and Leshan in Sichuan, where the two species may exhibit sympatric distribution.

### 2.2 Sequencing and assembly

#### 2.2.1 DNA extraction and sequencing

Genomic DNA was extracted from fresh samples using a whole-genome shotgun approach (Kahraman and Lucas, 2019) to construct libraries with insert sizes of approximately 400 base pairs (bp). Each library underwent paired-end (PE) sequencing utilizing second-generation sequencing (NGS) technology (Zheng et al., 2025) on the Illumina NovaSeq platform (Liu et al., 2022), with one library prepared per sample.

#### 2.2.2 Chloroplast genome assembly and annotation

Quality control of the raw sequencing data was performed using fastp (https://github.com/OpenGene/fastp) to filter and generate high-quality clean reads. The chloroplast genomes were then assembled using GetOrganelle v1.7.7.0 (Jin et al., 2020) (https://github.com/Kinggerm/GetOrganelle), resulting in complete circular chloroplast sequences.

As Amaranthus and Cyathula belong to the same family (Amaranthaceae), they share sufficient sequence similarity for read mapping. The plastid genome of Amaranthus tricolor possesses a conserved and well-annotated quadripartite structure (Mower and Vickrey, 2017; Viljoen et al., 2018). To ensure the accuracy of chloroplast genome assembly and annotation, the fully annotated sequence of Amaranthus tricolor (NC_065013) was retrieved from the National Center for Biotechnology Information (NCBI) database and used as the reference.Annotation of the 11 complete chloroplast genomes listed in **Supplementary Table 1** was performed using the online tool CPGAVAS2 (Shi et al., 2019) (http://47.96.249.172:16019/analyzer/annotate). The annotated features, including start and stop codons, introns, and exons, were manually verified to ensure accuracy. After confirming the correct annotation of protein-coding genes (CDs), rRNA genes, and tRNA genes, all sequencing data were submitted to the GenBank database at NCBI, with accession numbers provided in **Supplementary Table 1**. The CPGAVAS2 tool was also used to generate graphical maps of the annotated chloroplast genomes.

### 2.3 Basic characteristics and comparative analysis of chloroplast genomes

#### 2.3.1 Basic characteristics of chloroplast genomes

Genome-wide length, lengths of the LSC, SSC, and IR regions, as well as GC content, were statistically analyzed using Geneious Prime 2025.0.2 (Kearse et al., 2012).

#### 2.3.2 Analysis of simple sequence repeats and local sequence repeats in the chloroplast genome

SSRs in *Cyathula capitata*, *Cyathula officinalis*, and their hybrid (*Cyathula officinalis* × *Cyathula capitata*) were statistically analyzed using the online tool MISA (https://webblast.ipk-gatersleben.de/misa/) (Beier et al., 2017). The parameters were set as follows: mononucleotide repeats ≥10, dinucleotide repeats ≥6, trinucleotide repeats ≥5, and tetranucleotide, pentanucleotide, and hexanucleotide repeats ≥4.

Long repeat sequences were identified using the online software Reputer (https://bibiserv.cebitec.uni-bielefeld.de/reputer) (Kurtz and Schleiermacher, 1999), with a Hamming distance of 3 and a minimum repeat length of 30 bp. Four types of repeats were detected: forward, reverse, complementary, and palindromic repeats.

#### 2.3.3 Codon usage bias analysis

Protein-coding genes were extracted and screened from *Cyathula capitata*, *Cyathula officinalis*, and their hybrid (*Cyathula officinalis* × *Cyathula capitata*) using the following criteria: (1) sequences shorter than 300 bp were excluded; (2) sequences not beginning with the start codon ATG were removed; and (3) only one copy was retained for genes with multiple repeats. After filtering, the remaining gene sequences were merged, and the relative synonymous codon usage (RSCU) values were calculated using the formula: (RSCU = number of codons/total number of synonymous codons/number of synonymous codon types). Finally, codon usage preference analysis was conducted on the chloroplast genomes.

#### 2.3.4 Chloroplast genome comparative analysis

Variation analysis of the IR regions was conducted using IRscope software (https://irscope.shinyapps.io/irapp/) (Amiryousefi et al., 2018). Comparative and variation analyses of the chloroplast genomes of *Cyathula capitata*, *Cyathula officinalis*, and their hybrid (*Cyathula officinalis* × *Cyathula capitata*) were performed using mVISTA (https://genome.lbl.gov/vista/mvista/submit.shtml) (Frazer et al., 2004).

#### 2.3.5 Sequence variation analysis

Single-nucleotide polymorphisms (SNPs) represent sequence variations arising from single nucleotide changes, primarily through nucleotide transitions and transversions (Zou and Ge, 2003). To identify potential DNA barcode regions, nucleotide diversity (*Pi*) was calculated for both intergenic and coding regions to pinpoint SNP hotspots within *Cyathula capitata*, *Cyathula officinalis*, and their hybrid (*Cyathula officinalis* × *Cyathula capitata*). To identify highly variable regions, polymorphic sites and nucleotide diversity (Pi) in the six MAFFT-aligned CPGs were assessed using a sliding window analysis in DnaSP 6.10 (Yang et al., 2023).This analysis was complemented by mVISTA-based visual alignments of the chloroplast genomes.

#### 2.3.6 Phylogenetic analysis

In addition to the three target plants, we downloaded the complete chloroplast genome sequences of 15 other published Amaranthaceae species from NCBI, along with a white-flowered *Dianthus* species as an outgroup (**Table 1**). Multiple sequence alignment was performed with MAFFT v7.526 to optimize alignment accuracy and retain reliable phylogenetic signals (Zhang et al., 2020). Subsequently, the aligned sequences were entered into IQ-TREE v1.6.8, which was utilized to construct a phylogenetic tree based on the maximum likelihood method.

**Table 1:** Chloroplast genome information for phylogenetic inference.

Species name	Accession number
*Achyranthes* bidentata	MN652923
*Achyranthes* aspera	MN953051.1
*Achyranthes* longifolia	MN953049.1
*Amaranthus* tricolor	KX094399.1
*Alternanthera* philoxeroides	NC_0427989.1
*Celosia* cristata	NC045887
*Gomphrena* globosa	NC069834
*Suaeda* japonica	NC_02675.1
*Haloxylon* persicum	NC_027669.1
*Salsola* collina	NC066995
*Dysphania* botrys	NC_042166.1
*Dysphania* pumilio	NC_041159.1
*Oxybasis* glauca	NC_047226
*Chenopodium* ficifolium	NC_041200.1
*Chenopodium* quinoa	NC_034949
*Limonium* franchetii	OR374019
*Limonium* bicolor	NC_059915.1

## 3 Results

### 3.1 Basic characteristics of the chloroplast genomes of Naisibuni, *Cyathula officinalis*, and *Cyathula officinalis × Cyathula capitata*

The genome structures of Naisibuni, *Cyathula officinalis*, and the hybrid *Cyathula officinalis* × *Cyathula capitata* exhibit a typical closed quadripartite circular configuration, consistent with the chloroplast genome architecture of angiosperms. Analysis of the FASTA files revealed that sample C1 shares identical sequences with C2, Z1, Z3, Z5, and Z6; M1 is identical to Z2; while M2, M3, and Z4 each possess unique sequences. Notably, only C1, M1, M2, M3, and Z4 represent distinct sequence types. These results indicate that all collected *Cyathulae Radix* samples share identical sequences, whereas the three Naisibuni batches display sequence heterogeneity. Among the six batches of *Radix Achyranthis Bidentatae* var. *heterophylla*, only three distinct sequences were identified. Given that identical sequences share the same characteristics, subsequent analyses focused solely on these distinct sequence types.

**Figure 1:**
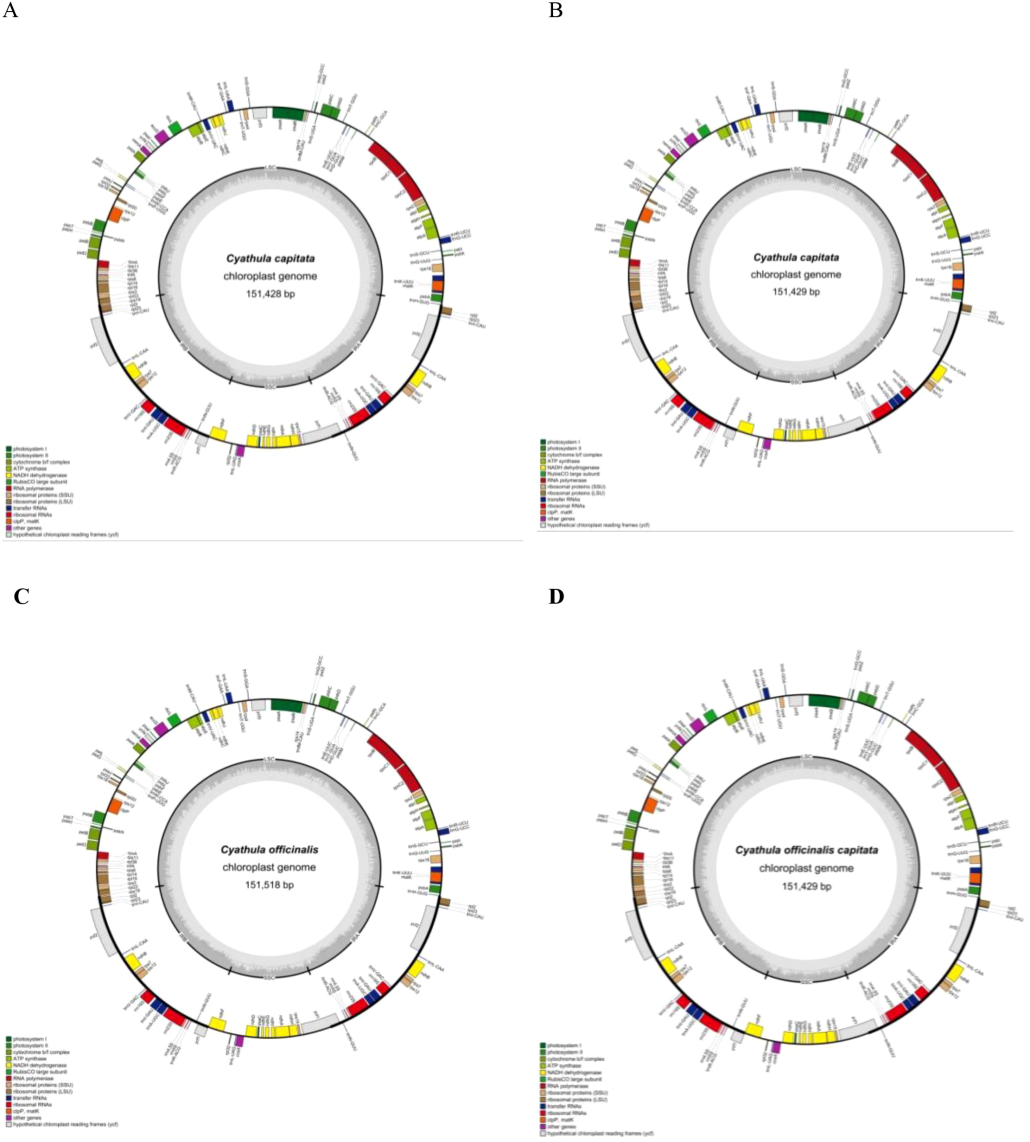
Chloroplast genome maps of C. *capitata* (Wall.) Moq. **(A, B)**, *C. officinalis* Kuan **(C)**, and C. officinalis*C. capitata **(D)**. Genes inside the circle are transcribed clockwise, and genes outside are transcribed counterclockwise. The dark gray inner circle corresponds to the GC content, and the light gray circle corresponds to the AT content.

The chloroplast genome of *Cyathula officinalis* spans 151,518 bp, comprising a pair of 25,229 bp IR regions separated by an 83,615 bp LSC region and a 17,445 bp SSC region. The complete chloroplast genome of *Cyathula capitata* ranges from 151,428 to 151,436 bp, containing the same 25,229 bp IR regions flanking an LSC region of 83,523–83,524 bp and an SSC region of 17,454–17,477 bp. For the hybrid *Cyathula officinalis* × *Cyathula capitata*, the chloroplast genome size varies from 151,429 to 151,518 bp, with 25,229 bp IR regions separating an LSC region of 83,524–83,615 bp and an SSC region of 17,445–17,477 bp (**Supplementary Table 2**). The GC content across all samples was consistent at 36.46%, with the highest GC content (42.51%) observed within the IR regions, exceeding the overall genome average. Annotation of the complete chloroplast genomes revealed 131 genes, including 86 protein-coding genes, 37 tRNA genes, and 8 rRNA genes (**Supplementary Table 3**). Among these, 15 genes contained a single intron, while three genes (*rps12*, *ycf3*, and *clpP*) contained two introns (**Supplementary Table 3**). Seventeen genes located within the IR regions were present in two copies, including *rps7*, *rps12*, *rpl2*, *rpl22*, *rrn4.5*, *rrn5*, *rrn16*, *rrn23*, *trnA-UGC*, *trnI-CAU*, *trnI-GAU*, *trnL-CAA*, *ycf1*, *ycf2*, *trnN-GUU*, *trnR-ACG*, and *trnV-GAC* (**Supplementary Table 3**). The annotated chloroplast genome sequences for all samples have been submitted to GenBank, with accession numbers listed in **Supplementary Table 1**. Gene maps illustrating the chloroplast genome structures for the different sequences are presented in **Figure 1**.

### 3.2 SSR and LSR analysis

SSRs, also known as microsatellites, are short tandem repeats consisting of 1 to 6 nucleotides. These sequences are characterized by the consecutive repetition of a small nucleotide motif, and the number, type, and distribution of SSR loci vary among species (Chen and Huang, 2004).

**Figure 2:**
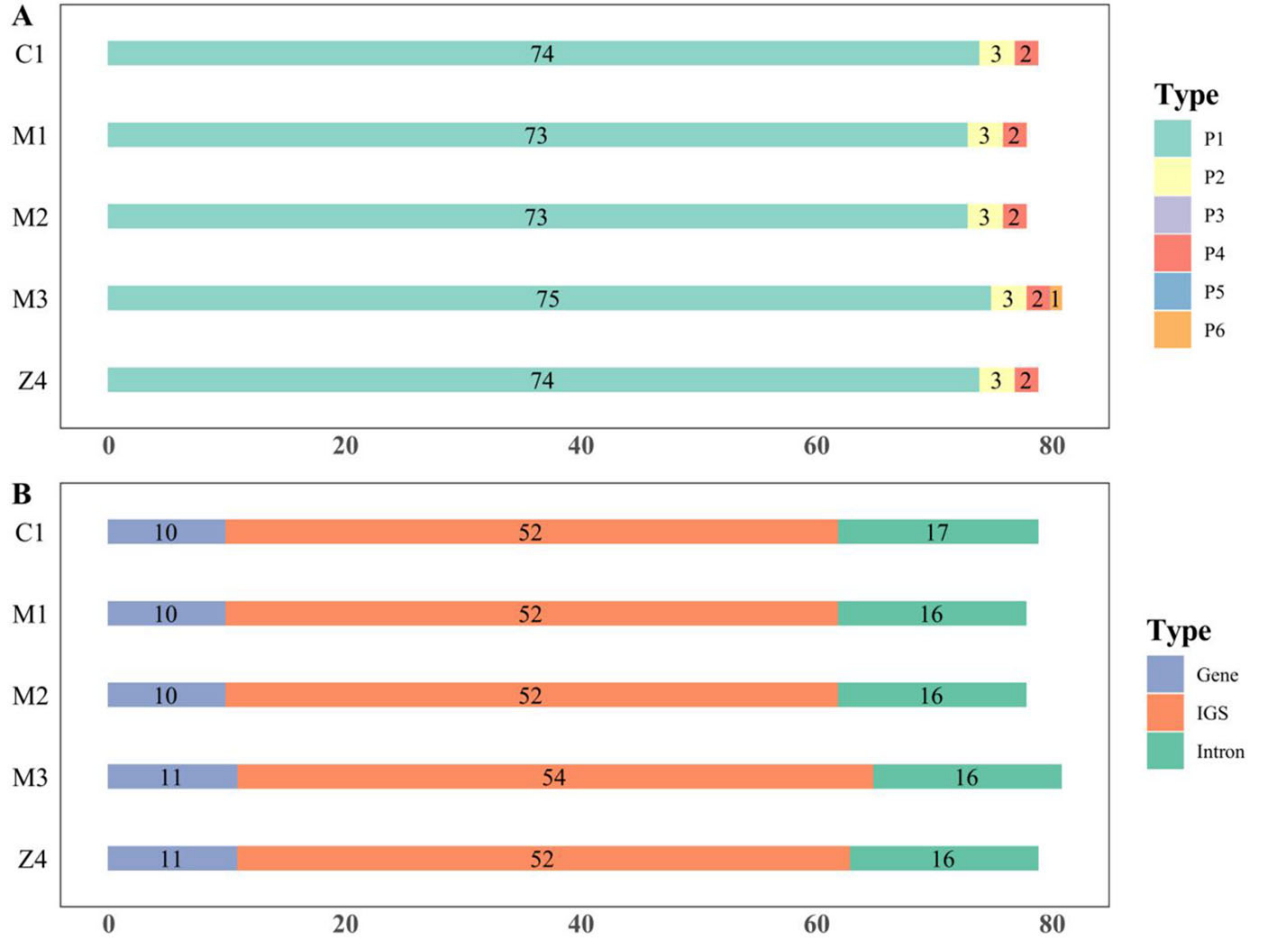
SSR distribution in the chloroplast genomes *of Cyathula capitata, Cyathula officinalis, and Cyathula officinalis × Cyathula capitata*. P1-Mononucleotide repeats P2-Dinucleotide repeats P3-Trinucleotide repeats. P4-Tetranucleotide repeats P5-Pentanucleotide repeats P6-Hexanucleotide repeats. **(A)** Nucleotide repeat types and counts **(B)** SSR distributions across genomic re.

Our analysis revealed that *Cyathula capitata* contains four types of SSRs: mononucleotide, dinucleotide, tetranucleotide, and hexanucleotide repeats. In contrast, *Cyathula officinalis* and the hybrid *Cyathula officinalis* × *Cyathula capitata* exhibit only three types: mononucleotide, dinucleotide, and tetranucleotide repeats. Mononucleotide repeats predominate across all three species, with T/A repeats being the most frequent. This prevalence aligns with previous research indicating that high AT content is a common feature of higher plant chloroplast genomes. SSRs are primarily distributed in IGS regions, predominantly within the LSC region of the chloroplast genome (Yang et al., 2019). While overall SSR characteristics are similar among the species, differences in the number and distribution of SSRs were observed (**Supplementary Table 4**, **Figure 2**). Specifically, mononucleotide SSRs accounted for 93.75–94.81% of SSRs in *Cyathula capitata*, with repeat lengths ranging from 10 to 15. In *Cyathula officinalis*, mononucleotide SSRs made up 94.87%, with lengths ranging from 10 to 16.

**Figure 3:**
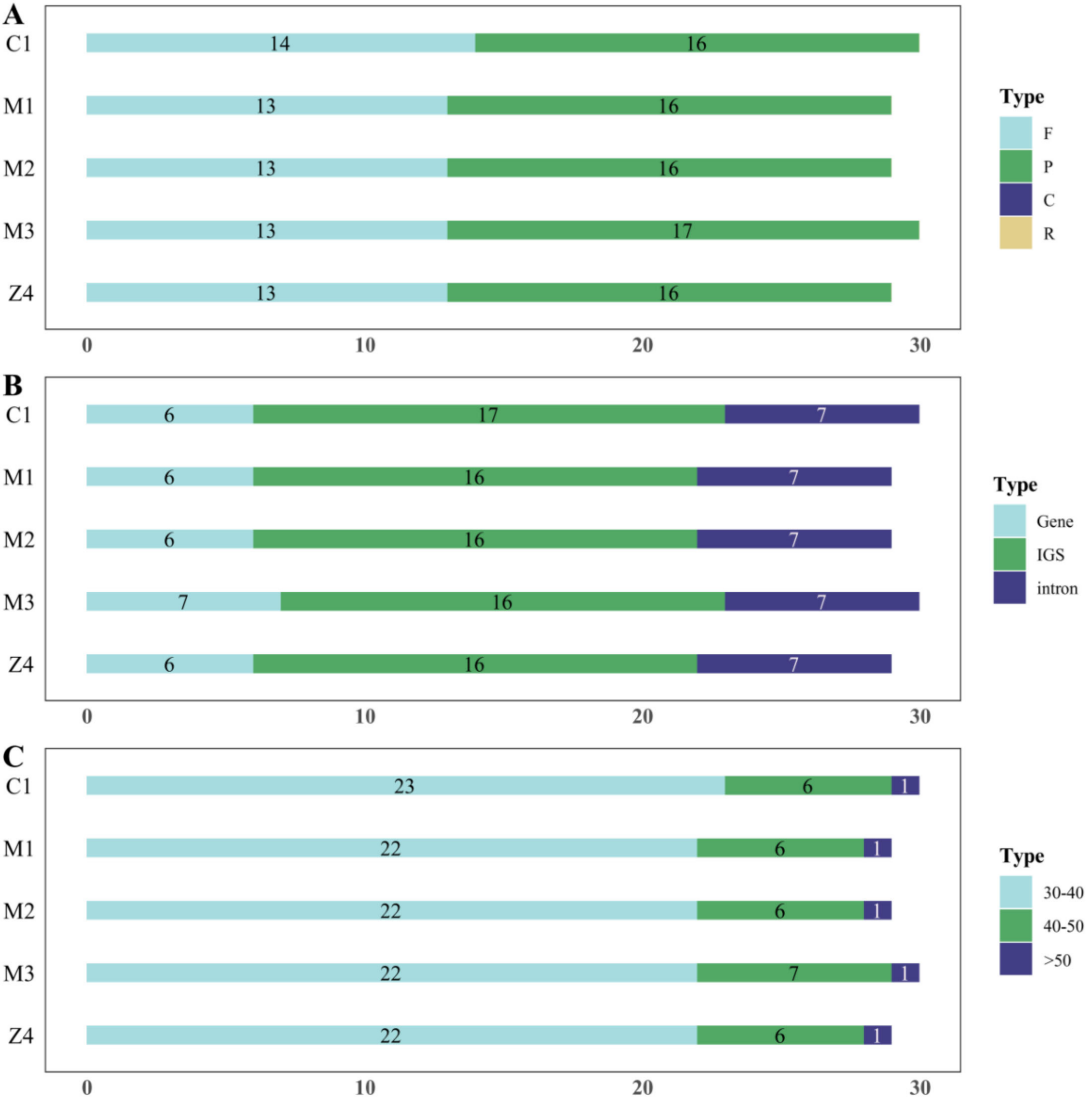
Distribution of LSRs in the chloroplast genomes of *Cyathula capitata, Cyathula officinalis, and Cyathula officinalis × Cyathula capitata*. F-Forward repeats P-Palindromic repeats C-Complementary repeats R-Reverse repeats. **(A)** Types of long repeats. **(B)** Genomic regions of LSR distribution. **(C)** Frequency of LSRs by length ranges.

The analysis of long repeat sequences (**Figure 3**) showed that all three species possess only palindromic and forward repeats, with palindromic repeats being more abundant. *Cyathula capitata* contained 29–30 long repeats, including 13 forward repeats; *Cyathula officinalis* had 30 total repeats with 14 forward repeats; and the hybrid exhibited 29–30 repeats with 13–14 forward repeats. These long repeats were primarily located within IGS regions. Notably, *Cyathula officinalis* contained an additional forward repeat within the *rps16_1-trnQ-UUG* intergenic region, ranging from 30 to 40 bp in length. Similarly, the M3 batch of the hybrid harbored an extra palindromic repeat within the *psbN* gene region, measuring 40 to 50 bp. Lastly, the Z1, Z3, Z5, and Z6 batches of the hybrid *Achillea* were identical to *Achillea chinensis*.

### 3.3 Codon usage bias analysis

**Figure 4:**
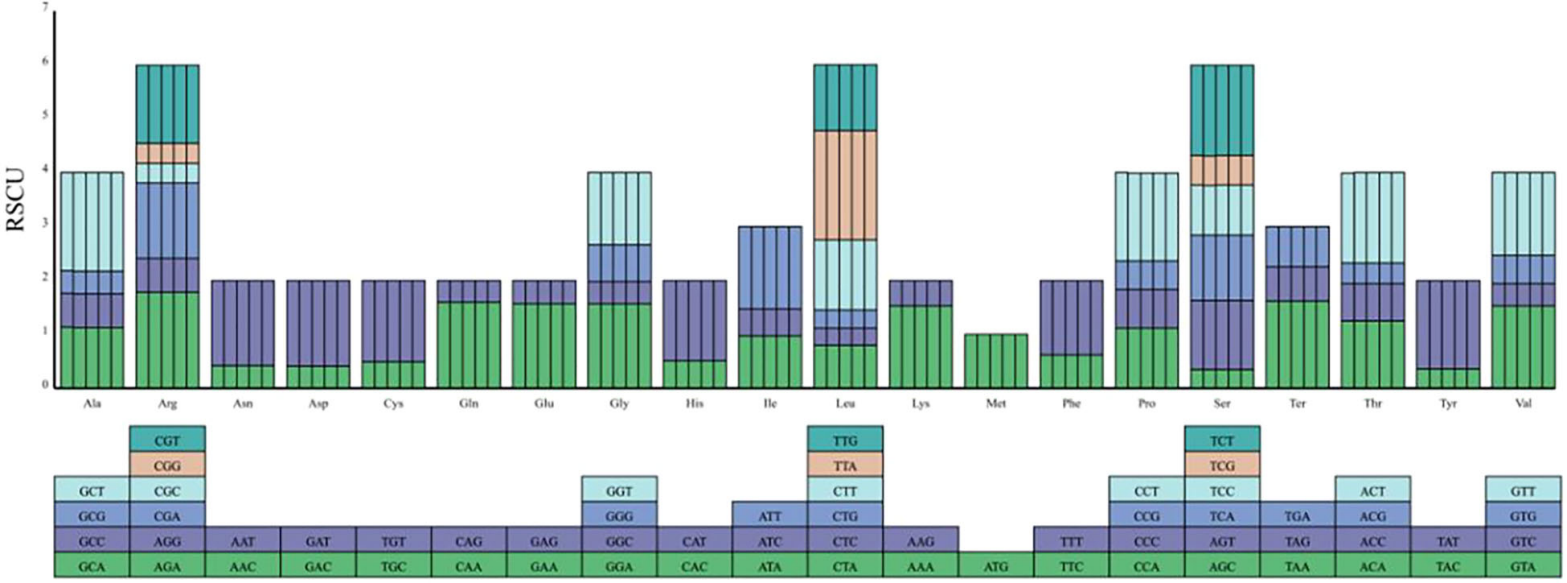
Codon usage frequency plots for *Cyathula capitata, Cyathula officinalis*, and *Cyathula officinalis × Cyathula capitata.* Amino acid bar charts from left to right: C1, M1, M2, M3, Z4.

The RSCU values for *Cyathula capitata, Cyathula officinalis, and their hybrid (Cyathula officinalis × Cyathula capitata*) (**Supplementary Table 5**), along with the synonymous codon relative abundance plots (**Figure 4**), reveal consistent patterns across all three species. The codons with the highest and lowest relative abundances are both associated with methionine (Met). Specifically, the codon GCT, encoding alanine (Ala), exhibited the highest relative abundance, while CTG, encoding leucine (Leu), showed the lowest. Although slight variations in relative codon usage frequencies were observed among the three chloroplast genomes, their overall codon usage patterns were largely similar. Codons ending with any of the four nucleotides (A, T, G, or C) displayed a usage frequency of approximately 16%. In *Cyathula officinalis* and *Cyathula capitata*, codons with differing relative usage frequencies, beyond GCA (Ala), were predominantly those ending with thymine (T). These included CCT (proline, Pro), GCT (Ala), and ACT (threonine, Thr). In *Cyathula capitata*, the only codon showing a distinctive relative usage frequency among T-ending codons was TCT, which encodes serine (Ser). As illustrated in **Figure 4** and **Supplementary Table 5**, all amino acids, except tryptophan encoded by TGG and Met encoded by ATG, are encoded by multiple codons. For example, arginine, Leu, and Ser each have six codons; Ala, glycine, Pro, Thr, and valine (Val) have four codons; isoleucine has three; and the remaining amino acids are encoded by two codons differing only at the third nucleotide position.

### 3.4 Comparison of IR region boundary positions in chloroplast genomes

**Figure 5:**
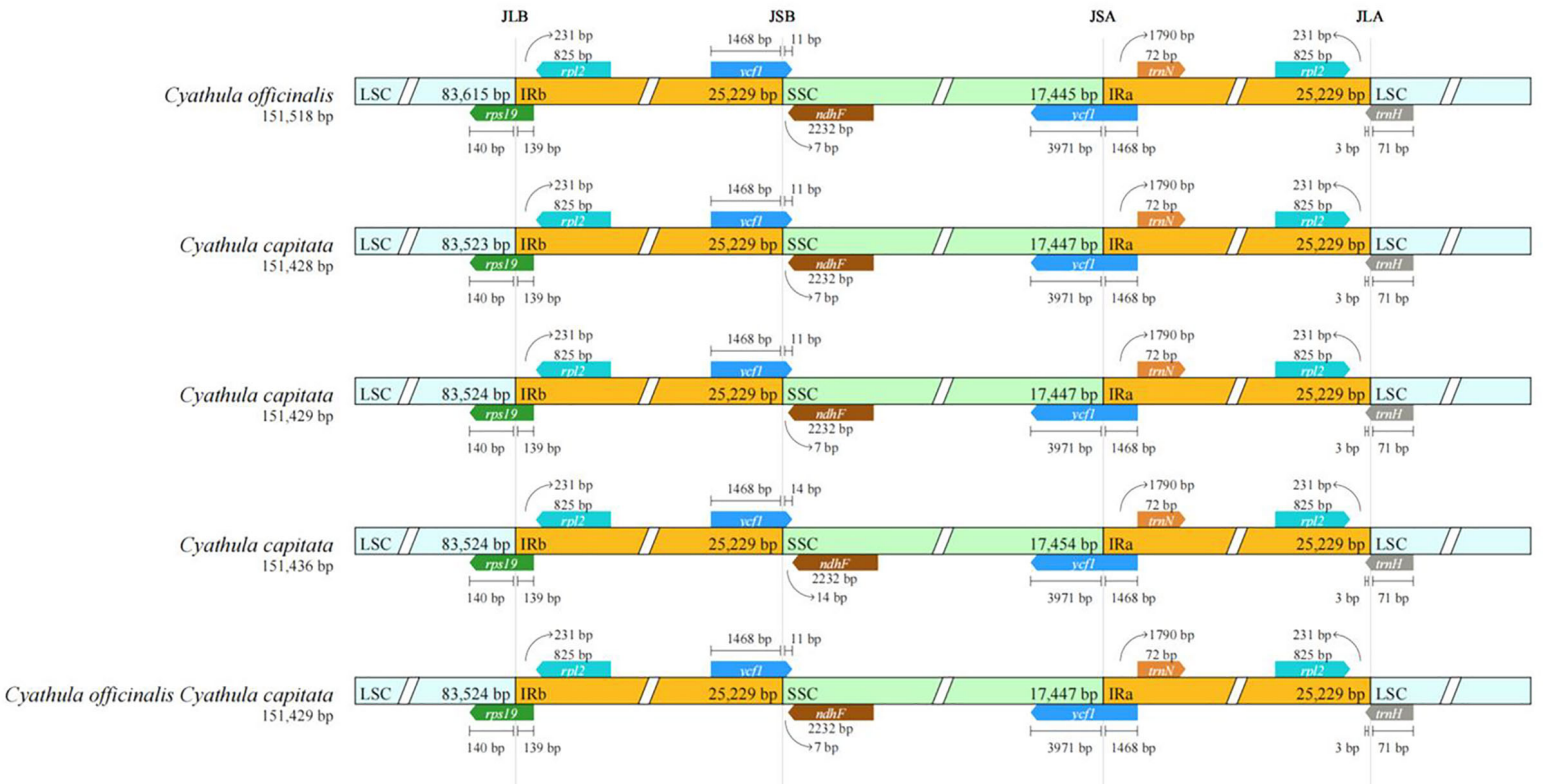
Comparison of IR boundary sequences in the chloroplast genomes of *Cyathula capitata*, *Cyathula officinalis*, and *Cyathula officinalis × Cyathula capitata*.

During the evolution of chloroplast genomes, contraction and expansion of boundary regions have frequently occurred (Zhang et al., 2024; Liu et al., 2023; Ju et al., 2017). The chloroplast genomes of *Cyathula capitata*, *Cyathula officinalis*, and their hybrid (*Cyathula officinalis* × *Cyathula capitata*) all exhibit the typical circular quadripartite structure, featuring four junctions: LSC–IRb, IRb–SSC, SSC–IRa, and IRa–LSC. Using the online tool IRscope, we compared the IR boundary regions across these species. The results (**Figure 5**) revealed that the LSC/IR and SSC/IR boundaries showed minimal variation among the three species. The SSC/IRa, IRa/LSC, and LSC/IRb junctions were highly conserved, with no observable differences. At the IRb/SSC boundary, the junction was located immediately adjacent to the *ndhF* gene in all species, except in *Cyathula capitata* (batch M3), where a 14 bp gap separated this boundary from the gene. These findings suggest that although the chloroplast genome boundaries among these species are relatively conserved with respect to gene content and sequence length at contraction and expansion sites, some variation still exist.

### 3.5 Analysis of chloroplast genome single nucleotide polymorphisms and sequence variability in plants

**Figure 6:**
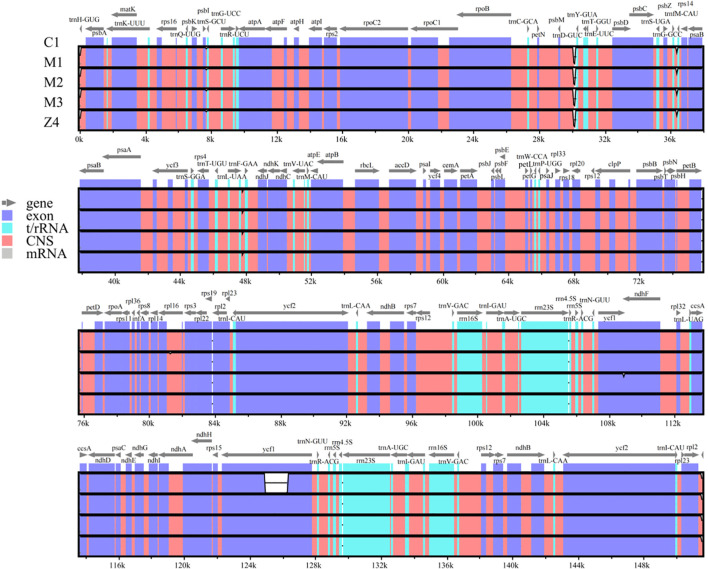
Complete chloroplast genome sequences of *Cyathula capitata*, *Cyathula officinalis, Cyathula officinalis* × *Cyathula capitata*. The vertical scale indicates the percentage of identity, ranging from 50% to 100%. The horizontal axis represents coordinates within the chloroplast genome. Genome regions are color-coded for protein-coding, rRNA, tRNA, intron, and conserved non-coding sequences.

Using the online software mVISTA, we analyzed sequence variation across the chloroplast genomes of the three species (**Figure 6**). The results confirmed that all three species belong to the Amaranthaceae family, exhibiting high overall sequence similarity while retaining certain distinct differences. Regions showing greater variability included *psbI-trnS-GCU*, *psbM-trnD-GUC*, *trnG-GCC-trnfM-CAU*, and *trnL-UAA-trnF-GAA*, with *trnH-GUG* identified as the most divergent gene. These hypervariable regions are promising candidates for developing specific molecular markers for species identification and phylogenetic studies.

**Figure 7:**
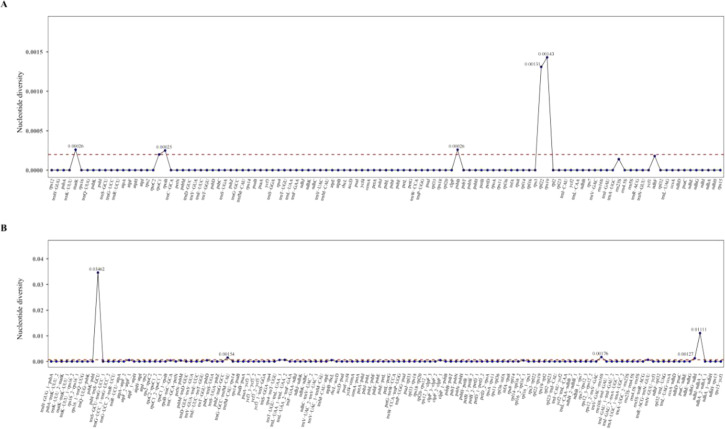
Nucleotide polymorphism analysis of chloroplast genomes in *Cyathula capitata*, *Cyathula officinalis*, and *Cyathula officinalis × Cyathula capitata.* **(A)** Coding regions **(B)** Intergenic regions.

To further characterize variation within these highly variable regions, nucleotide diversity (π) values were calculated for the chloroplast genomes of *Cyathula officinalis*, *Cyathula capitata*, and their hybrid (*Cyathula officinalis* × *Cyathula capitata*). Intergenic regions exhibited Pi values ranging from 0 to 0.03462 (**Figure 7B**), while coding regions showed lower variability, with Pi values ranging from 0 to 0.00143 (**Figure 7A**), indicating that coding sequences are relatively conserved across the three species. Among intergenic regions, the *psbI-trnS-GCU* interval displayed the highest nucleotide diversity (Pi = 0.03462). Located within the LSC region, this site represents a hypervariable zone and holds potential as a specific molecular marker for species identification. Within coding regions, the *rps19* gene exhibited the greatest nucleotide diversity (π = 0.00143). Since all π values for coding regions were below 0.0100, no coding genes displayed notably high variability.

### 3.6 Phylogenetic analysis

**Figure 8:**
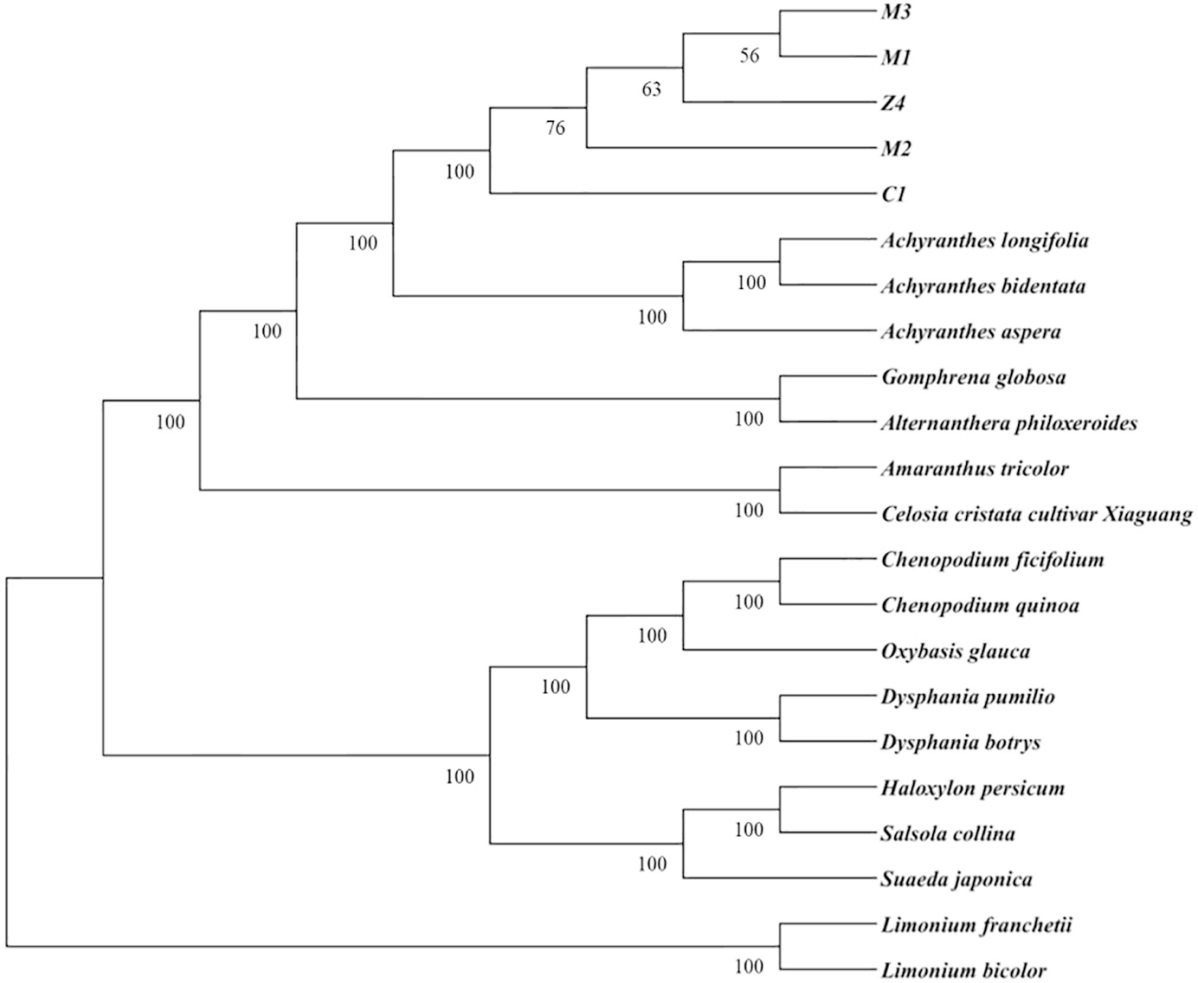
Phylogenetic tree based on complete chloroplast genome sequences.

The species from the *Plumbaginaceae* family constitute a distinct clade as the outgroup (**Figure 8**), while those from the *Amaranthaceae* family are grouped together and further divided into two major branches. One branch encompasses the genera *Chenopodium, Oxybasis*, *Dysphania*, *Salsola*, *Haloxylon*, and *Suaeda*; the other branch comprises the genera *Cyathula, Achyranthes, Gomphrena, Alternanthera, Amaranthus, and Celosia*. Among the species under consideration, those of *Achyranthes* and *Cyathula* exhibit the closest phylogenetic relationship. Within the genus *Cyathula*, *Cyathula officinalis* and *Cyathula capitata* form a monophyletic clade with 100% bootstrap support, and the two species are separated into distinct subclades. These findings indicate that phylogenetic analysis based on chloroplast genomes can clearly distinguish between them. The chloroplast genome of the hybrid (Z4) is nested within the *C. capitata* clade, which is consistent with the pattern of maternal chloroplast inheritance. That is, the offspring’s chloroplast genome is derived from the maternal parent. Therefore, the offspring’s chloroplast genome cannot be separated from the maternal lineage in the phylogenetic tree. Given the potential variability in maternal parents across different batches of the hybrid, the positions of these batches in the tree may also exhibit variation.

## 4 Discussion

Chloroplasts originated from free-living prokaryotes, but over time they have retained fewer genes from their ancestor, resulting in a progressive reduction in chloroplast genome size (Kleine et al., 2009). In angiosperms, chloroplasts are relatively conserved in structure, gene content, and gene arrangement (Xiao et al., 2025). Despite this overall conservation in structure and sequence, chloroplast genomes have been shown to vary in size, structure, and evolutionary rates of genes, even among closely related species (Williams et al., 2019). Due to features such as conserved architecture, lack of recombination, uniparental inheritance, and ease of amplification, chloroplast genomes play a significant role in phylogenetic and biogeographic studies (Song et al., 2024). Furthermore, chloroplast sequences are well-suited for high-throughput sequencing due to their high copy number, structural simplicity, highly conserved gene content, and relatively small size.With the ongoing decrease in the cost of second-generation sequencing technologies, efficient, rapid, and cost-effective high-quality sequencing of complete chloroplast genomes has become widely adopted (Ji et al., 2019). For instance, in genera such as *Scrophularia* and *Artemisia* L., comparative phylogenomic analyses based on chloroplast genomes have been effectively utilized for species delimitation and authentication of adulterants (Guo et al., 2023; Chen et al., 2024).

The *Amaranthaceae* family comprises approximately 60 genera and 850 species worldwide, with 13 genera and about 39 species recorded in China. Within this family, the genus *Cyathula* includes roughly 27 species distributed across Asia, Oceania, Africa, and the Americas. However, chloroplast genome data remain limited to only a few species, constraining our understanding of their evolutionary relationships and taxonomic classification.

Despite the evident variability among chloroplasts in terms of size, structure, and the rates at which genes evolve, the preservation of their structural and sequence characteristics is of paramount importance in the context of phylogenetic analysis and species identification (Yin et al., 2017). In this study, complete chloroplast genomes of *Cyathula capitata*, *C. officinalis*, and their hybrid were successfully obtained, thereby elucidating the structural features of their chloroplast genomes. The chloroplast genomes of *C. capitata, C. officinalis*, and the hybrid exhibited the characteristic closed circular quadripartite structure, with total lengths of 151,428–151,436 bp, 151,518 bp, and 151,429–151,518 bp, respectively. These lengths are comparable to those reported for *Corispermum patelliforme* (150,614 bp, 128 genes) (Yang et al., 2025), *Dysphania pumilio* (151,962 bp, 128 genes) (Kim et al., 2019), and other *Amaranthaceae* species (ranging from 149,726 to 153,474 bp based on NCBI data), aligning with the length characteristics observed in most angiosperm chloroplast genomes. Their complete chloroplast genomes consistently exhibited the typical quadripartite structure, consisting of the SSC, LSC, and two IR regions. Overall, the basic chloroplast genomic features of *C. capitata* and *C. officinalis* are consistent with those of most *Amaranthaceae* species, indicating high conservation in genome size and structure. The GC content was found to be higher in the IR regions compared to the LSC and SSC regions. This variation can be attributed to the presence of GC-rich genes within the IRs. Furthermore, while angiosperm chloroplast genomes typically exhibit an average GC content of approximately 35%, the three studied species demonstrated a slightly elevated GC content of 36.46%, which exceeds the typical average for angiosperms (Yang et al., 2025). The presence of introns within plant chloroplast genomes is a salient feature that merits close examination. In this study, it was found that *rps12*, *ycf3*, and *clpP* each contained two introns, while other intron-containing genes contained only one intron. These characteristics are consistent with those observed in the chloroplast genomes of most angiosperms.

Chloroplast SSR markers have been found to facilitate species identification due to their maternal inheritance (Wei et al., 2024)).SSR markers, known for their polymorphism, codominance, high reproducibility, and stability, are extensively applied in plant germplasm research (Drouin et al., 2008). We identified 79 SSRs in both *C. officinalis* and the hybrid. For *C. capitata*, batches M1 and M2 contained 78 SSRs each, while batch M3 exhibited 81 SSR loci. SSRs were predominantly located in the LSC region and mainly distributed within IGS regions of the single-copy regions across all three species. SSRs with repeat units of one to five nucleotides were present in all species, but hexanucleotide repeats were exclusive to *C. officinalis* and certain hybrid batches, likely reflecting maternal inheritance from *C. officinalis*. The *C. capitata* samples collected as Naisibuni lacked hexanucleotide repeats. Mononucleotide repeats reached a maximum length of 15 bp in *C. capitata* and some hybrids, while a unique 16 bp mononucleotide repeat was detected within the *rpl22* gene in *C. officinalis* and some hybrid batches. Additionally, an extra forward repeat sequence (30–40 bp) was identified in the *rps16_1-trnQ-UUG* region of *C. officinalis* and certain hybrid batches. These SSR characteristics may prove valuable for clarifying hybrid parental origins and developing precise molecular identification markers.

Codon usage bias analysis facilitates the discovery of novel genes and the prediction of gene function (Luo et al., 2024). In the three *Cyathula* species studied, thirty codons exhibited RSCU values greater than 1, indicating they are frequently used codons. Notably, 96.67% (29 out of 30) of these preferred codons ended with adenine (A) or uracil (U), reflecting a strong bias toward A- or U-ending codons. This bias correlates closely with the high AT content (63.54%) observed in the chloroplast genomes and suggests that an AT preference is a common feature within the genus *Cyathula*. These findings align well with previous codon preference analyses reported for 18 species in the Ampelopsideae tribe, four *Polygonatum* species (*Polygonatum odoratum* (Mill.) Druce, *Polygonatum sibiricum*, *Polygonatum cyrtonema*, and *Polygonatum kingianum*), and three *Astragalus* species (*Astragalus dahuricus* (Pall.) DC.,…).*Astragalus melilotoides Pall.* var. *tenuis Ledeb. and Astragalus laxmannii Jacq*.), where preferred codons similarly ended predominantly with A or U (Hu et al., 2024; Shi et al., 2024; Yin et al., 2024). The protein-coding sequences of the three *Cyathula* species comprise 64 codons, of which 61 encode the 20 standard amino acids, and the remaining three function as stop codons. Interestingly, the codons encoding Met exhibited both the smallest and largest relative abundance values. RSCU analysis revealed no strong bias at the third codon position among the three species, although codons with differing usage frequencies tended to end mostly with U. The results indicated that the chloroplast genomes of the three species were relatively conserved, with no significant differences observed in the IR boundaries. However, a specific variation was detected in the IRb/SSC boundary of the M3 batch of *Cyathula capitata*. Despite the genomic structure’s conservation at the species level, this finding points to the potential presence of low-frequency structural polymorphisms or minor intraspecific diversity within the population, thereby underscoring the biodiversity of this species.

*C. officinalis* × *C. capitata* is derived from *C. capitata* and *C. officinalis*. Given the maternal inheritance of the chloroplast genome, it is observed that certain hybrid samples exhibit characteristics indicative of the maternal parent. A comparative analysis of chloroplast genomes revealed variations among three batches of *C. capitata* collected from Sichuan, while *C. officinalis* samples from both Hubei and Sichuan provinces exhibited identical genotypes. These results suggest that the chloroplast genome of *C. capitata* exhibits genetic diversity, potentially influenced by geographical factors, while *C. officinalis* appears to exhibit a more conserved genetic profile. Among the six hybrid batches, over half exhibited genotypes identical to *C. officinalis*, suggesting a preferential maternal role of *C. officinalis* in hybridization. Preliminary research has demonstrated that the analysis of chloroplast gene sequencing data can yield ample information for the delineation of species and the reconstruction of evolutionary relationships (Bezeng et al., 2017; Li et al., 2023). Given the predominantly maternal inheritance of chloroplasts, several studies have demonstrated the utility of nuclear single-nucleotide polymorphism (SNP) markers in resolving plant phylogenies (Viljoen et al., 2018). Each chloroplast is subject to uniparental inheritance and is non-recombinant. A comparative analysis further indicated that regions of high divergence and hypervariable sites are primarily located within the LSC region. The *psbI-trnS-GCU* and *rps16_1-trnQ-UUG* intergenic spacers were identified as hypervariable loci. Furthermore, cluster analysis indicated that within the *Amaranthaceae* family, *C. officinalis* and *C. capitata* exhibit the strongest genetic affinity, constituting a monophyletic group with 100% bootstrap support. This finding serves to substantiate the reliability of the constructed phylogenetic tree. This outcome indicates that *C. officinalis* and *C. capitata* can be distinctly differentiated in the phylogeny that has been established from chloroplast genome sequences.

This study reports, for the first time, the complete chloroplast genomes of cultivated *Cyathula officinalis, C. capitata*, and *C. officinalis × C. capitata* from different production regions, thereby filling a significant research gap at the genomic level for these geographic germplasm resources. In summary, the findings of this study demonstrate a high degree of consistency with previous research across different plant groups. This consistency is evident in the analysis of chloroplast genome structure, codon usage patterns, and molecular identification techniques. The results suggest that these evolutionary and diagnostic patterns may be broadly applicable. This study provides a methodological foundation for authenticating “*C. officinalis*” in the market by analyzing the chloroplast genome information, phylogenetic relationships, and species identification of *C. capitata*.

## 5 Conclusions

This study sequenced, assembled, and annotated the chloroplast genomes of three *Cyathula capitata* samples collected from different regions. The chloroplast genomes ranged from 151,428 to 151,436 base pairs, comprising a pair of 25,229-bp IR regions separated by an 83,523–83,524 bp LSC region and a 17,454–17,477 bp SSC region. Comparative analyses with the chloroplast genomes of *Cyathula officinalis* and *Cyathula officinalis* var. *chinensis* provided further insights into their fundamental genomic features, including long and simple repetitive sequences, codon usage bias, and IR contraction and expansion patterns. Finally, a phylogenetic tree was constructed and analyzed, incorporating these three species along with 15 other published *Amaranthaceae* species. This study offers comprehensive information on the chloroplast genomes, phylogenetic relationships, and species identification of *Cyathula officinalis* and *Cyathula capitata*, establishing a reliable method for distinguishing *C. officinalis*, *C. capitata*, and their hybrid (*C. officinalis* × *C. capitata*) in the marketplace.

## Data Availability

The datasets presented in this study can be found in online repositories. The names of the repository/repositories and accession number(s) can be found in the article/**Supplementary Material**.

## Supplementary material

Supplementary Table 1 Sampling information and GenBank accession numbers for Naisibunia, Cyathula chinensis, and Cyathula heterophylla.

Supplementary Table 2 Basic Characteristics of Chloroplast Genomes.

Supplementary Table 3 Chloroplast genome genes of *Cyathula capitata*, *Cyathula officinalis*, and *Cyathula officinalis × Cyathula capitata.* () indicates number of gene copies; Gene* denotes one intron; Gene** denotes two introns.

Supplementary Table 4 SSR distribution in chloroplast genomes. IGS, CDS, and intron denote the intergenic region, protein-coding region, and intron region of the SSR within the chloroplast genome, respectively; LSC, IRa, SSC, and IRb represent the large single-copy region, inverted repeat region B, small single-copy region, and inverted repeat region A, respectively.

Supplementary Table 5 RSCU Values of *Cyathula capitata*, *Cyathula officinalis*, and *Cyathula officinalis* × *Cyathula capitata*.
