# Correction: Characteristic analysis of the chloroplast genome of *Cyathula capitata* and comparative analysis with *Cyathula officinalis* and their hybrid *Cyathula officinalis* × *Cyathula capitata*

**DOI:** 10.3389/fpls.2026.1825796

**Published:** 2026-05-06

**Authors:** Bixing Gao, Yisha Zeng, Min Tang, Maohua Yuan, Ke Deng, Yan Gou, Guihua Jiang

**Affiliations:** 1 Industrial Technology Basic Public Service Platform, Ministry of Industry and Information Technology, Sichuan Institute for Drug Control (Sichuan Medical Device Testing Center, Sichuan Musk Deer Research Institute), Chengdu, China; 2 College of Pharmacy, Chengdu University of Traditional Chinese Medicine, Chengdu, China; 3 Department of New Energy Materials and Chemistry, Leshan Normal University, Leshan, China

**Keywords:** chloroplast genome, *Cyathula capitata*, *Cyathula officinalis*, phylogenetic analysis, sequence characteristic

Affiliation 1 “Industrial Technology Basic Public Service Platform, Ministry of Industry and Information Technology, Sichuan Institute for Drug Control (Sichuan Medical Device Testing Center, Sichuan Musk Deer Research Institute), Chengdu, China” was erroneously given as “NMPA Key Laboratory for Quality Evaluation of Traditional Chinese Patent Medicine, Sichuan Institute for Drug Control, Chengdu, China”.

The original version of this article has been updated.

