# Supplementary material for: Characteristic analysis of the chloroplast genome of Cyathula capitata and comparative analysis with Cyathula officinalis and their hybrid Cyathula officinalis × Cyathula capitata
Source: Front Plant Sci. 2026 Mar 4;17:1772997. doi: 10.3389/fpls.2026.1772997 (PMC12997787; doi:10.3389/fpls.2026.1772997)
Supplement: Supplementary Table 1 — Sampling information and GenBank accession numbers for Naisibunia, Cyathula chinensis, and Cyathula heterophylla. [file Table1.docx]

**Table S 1 Sampling information and GenBank accession numbers for Naisibunia, Cyathula chinensis, and Cyathula heterophylla**

| Species Latin Name | Chinese Name | Herbal Name | Number | Location | Longitude | Latitude | Elevation | | Registration Number |
| --- | --- | --- | --- | --- | --- | --- | --- | --- | --- |
| *Cyathula capitata* (Wall.) Moq. | *Cyathula capitata* | Naisbuni | M1 | Longdong Town, Donglashan Grand Canyon, Baoxing County, Ya'an City, Sichuan Province | 102°57′55″E | 30°38′56″N | 3773.10 | PV138967 | |
|  |  |  | M2 | Zhangmu Village, Taiping Town, Huili City, Liangshan Yi Autonomous Prefecture, Sichuan Province | 102°24′9″E | 26°45′56″N | 2036.50 | PV138968 | |
|  |  |  | M3 | Namajiu Village, Teguo Township, Puge County, Liangshan Yi Autonomous Prefecture, Sichuan Province | 102°27′20″E | 27°41′8″N | 2309.00 | PV138969 | |
| *Cyathula officinalis* Kuan | Chuan Niuxi | Chuan Niuxi | C1 | Yanbanhe, Guandian Town, Jianshi County, Enshi Tujia and Miao Autonomous Prefecture, Hubei Province | 110°2′46″E | 30°8′54″N | 1904.90 | PV138959 | |
|  |  |  | C2 | Shunhua Highway, Duowoping, Yongsheng Township, Jinkouhe District, Leshan City, Sichuan Province | 103°1′46″E | 29°21′35″N | 1404.30 | PV138960 | |
| C. officinalis × C. capitata | Hybrid Achyranthes | Hybrid Achyranthes | Z1 | Longdong Town, Donglashan Grand Canyon, Baoxing County, Ya'an City, Sichuan Province | 102°57′58″E | 30°38′52″N | 3778.50 | PV138961 | |
|  |  |  | Z2 | Longdong Town, Donglashan Grand Canyon, Baoxing County, Ya'an City, Sichuan Province | 102°57′55″E | 30°38′56″N | 3770.00 | PV138962 | |
|  |  |  | Z3 | , Dongla Mountain Grand Canyon, Baoxing County, Ya'an City, Sichuan Province Longdong Town | 102°57′54″E | 30°38′55″N | 3776.00 | PV138963 | |
|  |  |  | Z4 | Hazi Village, Pianma Yi Ethnic Township, Hanyuan County, Ya'an City, Sichuan Province | 102°43′47″E | 29°14′38″N | 2135.20 | PV138964 | |
|  |  |  | Z5 | Hazi, Pianma Yi Ethnic Township, Hanyuan County, Ya'an City, Sichuan Province | 102°43′45″E | 29°14′32″N | 2097.60. | PV138965 | |
|  |  |  | Z6 | Shunhua Highway, Duowoping, Yongsheng Township, Jinkouhe District, Leshan City, Sichuan Province | 103°1′45″E | 29°21′33″N | 1400.30 | PV138966 | |

**Table S 2 Basic Characteristics of Chloroplast Genomes**

|  | M1 | M2 | M3 | C1 | Z4 |
| --- | --- | --- | --- | --- | --- |
| Gene Length (bp) | 151428 | 151429 | 151436 | 151518 | 151429 |
| LSC Region Length (bp) | 83523 | 83524 | 83524 | 83615 | 83524 |
| SSC region length (bp) | 17447 | 17447 | 17454 | 17445 | 17447 |
| IR region length (bp) | 25229 | 25229 | 25229 | 25229 | 25229 |
| Number of protein-coding genes | 86 | 86 | 86 | 86 | 86 |
| tRNA quantity | 37 | 37 | 37 | 37 | 37 |
| rRNA quantity | 8 | 8 | 8 | 8 | 8 |
| Total GC Content (%) | 36.46 | 36.46 | 36.46 | 36.46 | 36.46 |
| LSC Zone GC Content (%) | 34.19 | 34.19 | 34.19 | 34.19 | 34.19 |
| SSC Zone GC Content (%) | 29.80 | 29.81 | 29.80 | 29.81 | 29.81 |
| IR Zone GC Content (%) | 42.51 | 42.51 | 42.51 | 42.51 | 42.51 |

**Table S 3 Chloroplast genome genes of *Cyathula capitata*, *Cyathula officinalis*, and**

***Cyathula officinalis × Cyathula capitata***

| Classification | Genome | Gene Name |
| --- | --- | --- |
| Photosynthesis-related genes | Photosystem I | *psaA, psaB, psaC, psaI, psaJ* |
|  | Photosystem II | *psbA, psbB, psbC, psbD, psbE, psbF, psbH, psbI, psbJ, psbK, psbL, psbM, psbN, psbT, psbZ* |
|  | cytochrome b/f complex | *petA, petB*, petD*, petG, petL, petN* |
|  | ATP synthase | *atpA, atpB, atpE, atpF*, atpH, atpI* |
|  | NADH dehydrogenase | *ndhA*, ndhB*, ndhC, ndhD, ndhE, ndhF, ndhG, ndhH, ndhI, ndhJ, ndhK* |
|  | RubisCO large subunit | *rbcL* |
| Replication genes | RNA polymerase | *rpoA, rpoB, rpoC1*, rpoC2* |
|  | ribosomal proteins (SSU) | *rps2, rps3, rps4, rps7 (2), rps8, rps11, rps12** (2), rps14, rps15, rps16*, rps18, rps19* |
|  | ribosomal proteins (LSU) | *rpl2 (2), rpl14, rpl16*, rpl20, rpl22 (2), rpl23, rpl32, rpl33, rpl36* |
|  | transfer RNAs | *trnA-UGC* (2), trnC-GUG, trnD-GUC, trnE-UUC, trnF-GAA, trnG-GCC, trnG-UCC*, trnH-GUG*, trnH-GCA, trnI-CAU (2), trnI-GAU* (2), trnK-UUU*, trnL-UAA*, trnL-CAA (2), trnL-UAG, trnM-CAU, trnfM-CAU, trnN-GUU (2), trnP-UGG, trnQ-UUG, trnR-UCU, trnR-ACG (2), trnS-UGA, trnS-GGA, trnS-GCU, trnT-UGU, trnT-GGU, trnV-UAC*, trnV-GAC (2), trnW-CCA, trnY-GUA* |
|  | ribosomal RNAs | *rrn4.5s (2), rrn5s (2), rrn16s (2), rrn23s (2)* |
| Other Genes | Proteases | *clpP*** |
|  | Mature enzymes | *matK* |
|  | cytochrome c synthesis gene | *ccsA* |
|  | Envelope membrane protein | *cemA* |
|  | Deaminase | *accD* |
| Function-unknown gene | Conserved open reading frame | *ycf1 (2), ycf2 (2), ycf3, ycf4*** |

( ) indicates number of gene copies; Gene* denotes one intron; Gene** denotes two introns

**Table S 4 SSR distribution in chloroplast genomes**

| Number | Repeat Unit | requency per region/unit | | | Frequency per Position/Unit | | | | Frequency by Length/Unit | | | | | | | | | Total |
| --- | --- | --- | --- | --- | --- | --- | --- | --- | --- | --- | --- | --- | --- | --- | --- | --- | --- | --- |
|  |  | IGS | Intron | Gene | LSC | IRb | SSC | IRa | 4 | 6 | 10 | 11 | 12 | 13 | 14 | 15 | 16 |  |
| M1 | A | 18 | 6 | 1 | 18 | 2 | 4 | 1 |  |  | 7 | 8 | 5 | 3 | 2 |  |  | 25 |
|  | T | 28 | 9 | 9 | 36 | 1 | 7 | 2 |  |  | 17 | 13 | 9 | 3 | 3 | 1 |  | 46 |
|  | G | 1 |  |  |  | 1 |  |  |  |  | 1 |  |  |  |  |  |  | 1 |
|  | C | 1 |  |  |  |  |  | 1 |  |  | 1 |  |  |  |  |  |  | 1 |
|  | AT | 1 |  |  | 1 |  |  |  |  | 1 |  |  |  |  |  |  |  | 1 |
|  | TA | 2 |  |  | 2 |  |  |  |  | 2 |  |  |  |  |  |  |  | 2 |
|  | TAAT | 1 |  |  | 1 |  |  |  | 1 |  |  |  |  |  |  |  |  | 1 |
| M2 | A | 18 | 6 | 1 | 18 | 2 | 4 | 1 |  |  | 7 | 8 | 4 | 4 | 2 |  |  | 25 |
|  | T | 28 | 9 | 9 | 36 | 1 | 7 | 2 |  |  | 18 | 12 | 9 | 3 | 3 | 1 |  | 46 |
|  | G | 1 |  |  |  | 1 |  |  |  |  | 1 |  |  |  |  |  |  | 1 |
|  | C | 1 |  |  |  |  |  | 1 |  |  | 1 |  |  |  |  |  |  | 1 |
|  | AT | 1 |  |  | 1 |  |  |  |  | 1 |  |  |  |  |  |  |  | 1 |
|  | TA | 2 |  |  | 2 |  |  |  |  | 2 |  |  |  |  |  |  |  | 2 |
|  | TAAT | 1 |  |  | 1 |  |  |  | 1 |  |  |  |  |  |  |  |  | 1 |
| M3 | A | 19 | 6 | 2 | 20 | 2 | 4 | 1 |  |  | 8 | 8 | 5 | 4 | 2 |  |  | 27 |
|  | T | 28 | 9 | 9 | 36 | 1 | 7 | 2 |  |  | 17 | 13 | 10 | 2 | 3 | 1 |  | 46 |
|  | G | 1 |  |  |  | 1 |  |  |  |  | 1 |  |  |  |  |  |  | 1 |
|  | C | 1 |  |  |  |  |  | 1 |  |  | 1 |  |  |  |  |  |  | 1 |
|  | AT | 1 |  |  | 1 |  |  |  |  | 1 |  |  |  |  |  |  |  | 1 |
|  | TA | 2 |  |  | 2 |  |  |  |  | 2 |  |  |  |  |  |  |  | 2 |
|  | TAAT | 1 |  |  | 1 |  |  |  | 1 |  |  |  |  |  |  |  |  | 1 |
|  | AAATAG | 1 |  |  | 1 |  |  |  | 1 |  |  |  |  |  |  |  |  | 1 |
| C1 | A | 19 | 6 | 1 | 19 | 2 | 4 | 1 |  |  | 9 | 6 | 4 | 5 | 2 |  |  | 26 |
|  | T | 27 | 10 | 9 | 36 | 1 | 7 | 2 |  |  | 17 | 14 | 8 | 3 | 2 | 1 | 1 | 46 |
|  | G | 1 |  |  |  | 1 |  |  |  |  | 1 |  |  |  |  |  |  | 1 |
|  | C | 1 |  |  |  |  |  | 1 |  |  | 1 |  |  |  |  |  |  | 1 |
|  | AT | 1 |  |  | 1 |  |  |  |  | 1 |  |  |  |  |  |  |  | 1 |
|  | TA | 2 |  |  | 2 |  |  |  |  | 2 |  |  |  |  |  |  |  | 2 |
|  | TAAT | 1 |  |  | 1 |  |  |  | 1 |  |  |  |  |  |  |  |  | 1 |
| Z4 | A | 18 | 6 | 2 | 19 | 2 | 4 | 1 |  |  | 7 | 8 | 5 | 4 | 2 |  |  | 26 |
|  | T | 28 | 9 | 9 | 36 | 1 | 7 | 2 |  |  | 17 | 13 | 9 | 3 | 3 | 1 |  | 46 |
|  | G | 1 |  |  |  | 1 |  |  |  |  | 1 |  |  |  |  |  |  | 1 |
|  | C | 1 |  |  |  |  |  | 1 |  |  | 1 |  |  |  |  |  |  | 1 |
|  | AT | 1 |  |  | 1 |  |  |  |  | 1 |  |  |  |  |  |  |  | 1 |
|  | TA | 2 |  |  | 2 |  |  |  |  | 2 |  |  |  |  |  |  |  | 2 |
|  | TAAT | 1 |  |  | 1 |  |  |  | 1 |  |  |  |  |  |  |  |  | 1 |

IGS, CDS, and intron denote the intergenic region, protein-coding region, and intron region of the SSR within the chloroplast genome, respectively; LSC, IRa, SSC, and IRb represent the large single-copy region, inverted repeat region B, small single-copy region, and inverted repeat region A, respectively.

**Table S 5 RSCU Values of Cyathula capitata, Cyathula officinalis, and Cyathula officinalis × Cyathula capitata**

| Amino acid | Codon | N0 | C1-RSCU | M1-RSCU | M2-RSCU | M3-RSCU | Z4-RSCU |
| --- | --- | --- | --- | --- | --- | --- | --- |
| Trp | TGG | 380 | 1 | 1 | 1 | 1 | 1 |
| Gly | GGT | 474 | 1.34 | 1.34 | 1.34 | 1.34 | 1.34 |
| Arg | AGG | 130 | 0.63 | 0.63 | 0.63 | 0.63 | 0.63 |
| Arg | CGA | 287 | 1.4 | 1.4 | 1.4 | 1.4 | 1.4 |
| Cys | TGT | 175 | 1.51 | 1.51 | 1.51 | 1.51 | 1.51 |
| Gly | GGG | 240 | 0.68 | 0.68 | 0.68 | 0.68 | 0.68 |
| Arg | AGA | 365 | 1.78 | 1.78 | 1.78 | 1.78 | 1.78 |
| Arg | CGC | 73 | 0.36 | 0.36 | 0.36 | 0.36 | 0.36 |
| Cys | TGC | 57 | 0.49 | 0.49 | 0.49 | 0.49 | 0.49 |
| Gly | GGA | 555 | 1.57 | 1.57 | 1.57 | 1.57 | 1.57 |
| Ser | AGC | 91 | 0.35 | 0.35 | 0.35 | 0.35 | 0.35 |
| Arg | CGG | 76 | 0.37 | 0.37 | 0.37 | 0.37 | 0.37 |
| Tyr | TAC | 141 | 0.36 | 0.36 | 0.36 | 0.36 | 0.36 |
| Gly | GGC | 146 | 0.41 | 0.41 | 0.41 | 0.41 | 0.41 |
| Ser | AGT | 329 | 1.28 | 1.28 | 1.28 | 1.28 | 1.28 |
| Arg | CGT | 298 | 1.45 | 1.45 | 1.45 | 1.45 | 1.45 |
| Tyr | TAT | 642 | 1.64 | 1.64 | 1.64 | 1.64 | 1.64 |
| Asp | GAT | 667 | 1.59 | 1.59 | 1.59 | 1.59 | 1.59 |
| Lys | AAG | 266 | 0.47 | 0.47 | 0.47 | 0.47 | 0.47 |
| Gln | CAA | 630 | 1.6 | 1.6 | 1.6 | 1.6 | 1.6 |
| Ser | TCT | 430 | 1.67 | 1.68 | 1.67 | 1.67 | 1.67 |
| Asp | GAC | 172 | 0.41 | 0.41 | 0.41 | 0.41 | 0.41 |
| Lys | AAA | 863 | 1.53 | 1.53 | 1.53 | 1.53 | 1.53 |
| Gln | CAG | 158 | 0.4 | 0.4 | 0.4 | 0.4 | 0.4 |
| Ser | TCG | 142 | 0.55 | 0.55 | 0.55 | 0.55 | 0.55 |
| Glu | GAA | 884 | 1.57 | 1.57 | 1.57 | 1.57 | 1.57 |
| Asn | AAC | 216 | 0.42 | 0.42 | 0.42 | 0.42 | 0.42 |
| His | CAT | 365 | 1.49 | 1.49 | 1.49 | 1.49 | 1.49 |
| Ser | TCA | 311 | 1.21 | 1.21 | 1.21 | 1.21 | 1.21 |
| Glu | GAG | 244 | 0.43 | 0.43 | 0.43 | 0.43 | 0.43 |
| Asn | AAT | 803 | 1.58 | 1.58 | 1.58 | 1.58 | 1.58 |
| His | CAC | 126 | 0.51 | 0.51 | 0.51 | 0.51 | 0.51 |
| Ser | TCC | 238 | 0.93 | 0.92 | 0.93 | 0.93 | 0.93 |
| Ala | GCA | 324 | 1.14 | 1.13 | 1.13 | 1.13 | 1.13 |
| Thr | ACC | 181 | 0.69 | 0.69 | 0.69 | 0.69 | 0.69 |
| Pro | CCT | 352 | 1.64 | 1.63 | 1.63 | 1.63 | 1.63 |
| Phe | TTT | 820 | 1.38 | 1.38 | 1.38 | 1.38 | 1.38 |
| Ala | GCC | 176 | 0.62 | 0.62 | 0.62 | 0.62 | 0.62 |
| Thr | ACA | 327 | 1.25 | 1.25 | 1.25 | 1.25 | 1.25 |
| Pro | CCG | 115 | 0.53 | 0.53 | 0.53 | 0.53 | 0.53 |
| Phe | TTC | 365 | 0.62 | 0.62 | 0.62 | 0.62 | 0.62 |
| Ala | GCG | 121 | 0.42 | 0.42 | 0.42 | 0.42 | 0.42 |
| Thr | ACG | 99 | 0.38 | 0.38 | 0.38 | 0.38 | 0.38 |
| Pro | CCA | 242 | 1.12 | 1.12 | 1.12 | 1.12 | 1.12 |
| Leu | TTG | 445 | 1.22 | 1.22 | 1.22 | 1.22 | 1.22 |
| Ala | GCT | 520 | 1.82 | 1.83 | 1.83 | 1.83 | 1.83 |
| Thr | ACT | 437 | 1.67 | 1.68 | 1.68 | 1.68 | 1.68 |
| Pro | CCC | 152 | 0.71 | 0.71 | 0.71 | 0.71 | 0.71 |
| Leu | TTA | 739 | 2.03 | 2.03 | 2.03 | 2.03 | 2.03 |
| Val | GTA | 433 | 1.53 | 1.53 | 1.53 | 1.53 | 1.53 |
| Ile | ATC | 297 | 0.5 | 0.5 | 0.5 | 0.5 | 0.5 |
| Leu | CTA | 291 | 0.8 | 0.8 | 0.8 | 0.8 | 0.8 |
| Ter | TAG | 11 | 0.63 | 0.63 | 0.63 | 0.63 | 0.63 |
| Val | GTC | 116 | 0.41 | 0.41 | 0.41 | 0.41 | 0.41 |
| Ile | ATA | 580 | 0.97 | 0.97 | 0.97 | 0.97 | 0.97 |
| Leu | CTT | 472 | 1.3 | 1.3 | 1.3 | 1.3 | 1.3 |
| Ter | TGA | 13 | 0.75 | 0.75 | 0.75 | 0.75 | 0.75 |
| Val | GTG | 150 | 0.53 | 0.53 | 0.53 | 0.53 | 0.53 |
| Ile | ATT | 918 | 1.53 | 1.53 | 1.53 | 1.53 | 1.53 |
| Leu | CTC | 117 | 0.32 | 0.32 | 0.32 | 0.32 | 0.32 |
| Ter | TAA | 28 | 1.62 | 1.62 | 1.62 | 1.62 | 1.62 |
| Val | GTT | 431 | 1.53 | 1.53 | 1.53 | 1.53 | 1.53 |
| Met | ATG | 465 | 1 | 1 | 1 | 1 | 1 |
| Leu | CTG | 120 | 0.33 | 0.33 | 0.33 | 0.33 | 0.33 |
